# Selection of reference genes for tissue/organ samples of adults of *Eucryptorrhynchus scrobiculatus*

**DOI:** 10.1371/journal.pone.0228308

**Published:** 2020-02-03

**Authors:** Peng Gao, Jingzhen Wang, Junbao Wen

**Affiliations:** Beijing Key Laboratory for Forest Pest Control, Beijing Forestry University, Beijing, P. R. China; Northwestern University Feinberg School of Medicine, UNITED STATES

## Abstract

*Eucryptorrhynchus scrobiculatus* is an important wood-boring pest of *Ailanthus altissima* in China, where it causes a large number of these trees to weaken or even die. To identify genes related to economic traits or specific cellular processes in *E*. *scrobiculatus*, gene expression in multiple tissue/organ samples is commonly surveyed, and reference genes are required in this process as a control for normalization. In the present study, 18 candidate reference genes from *E*. *scrobiculatus* were identified, and the expression levels of these reference genes were estimated through quantitative real-time PCR. Differences in expression levels were analyzed with four algorithms (geNorm, NormFinder, BestKeeper and delta Ct) and comprehensively with RefFinder. With the most stable levels of expression in different tissues, RPL13, RPS3 and RPL36 were determined to be suitable for use as candidate reference genes. Moreover, the expression profile of one target gene (glycoside hydrolase family 45, GH45) confirmed the reliability of the selected candidate reference genes. This study provides the first set of suitable candidate reference genes for gene expression studies in *E*. *scrobiculatus*, and the findings will facilitate subsequent transcriptomics and functional gene research on this pest.

## 1. Introduction

*Eucryptorrhynchus scrobiculatus* Motschulsky (Coleoptera: Curculionidae), an important wood-boring pest of the tree-of-heaven *Ailanthus altissima* Swingl [1–3] that is widely distributed in 21 provinces in China [4], is one of the most damaging invasive insects in this country. *E*. *scrobiculatus* larvae feed on *A*. *altissima* roots, and adults feed on *A*. *altissima* twigs, which usually kills the trees within 3–5 years [5]. Considered an invasive species, *A*. *altissima* is a serious threat to ecosystems in North America; as *E*. *scrobiculatus* specifically feeds on this tree, it has great promise as a potential biological control agent [1, 6]. To study the biology of *E*. *scrobiculatus*, it is important to identify plant cell wall-degrading enzymes and functional genes involved in host-parasite interactions; however, reference genes must first be selected. Fortunately, *E*. *scrobiculatus* transcriptomes are available and provide comprehensive information to facilitate such studies.

Gene expression analysis can be used to identify genes related to biological processes and physiological conditions [2]. Real-time quantitative RT-PCR (RT-qPCR) is the most commonly used technology for accurately detecting gene expression, and it has been widely applied in molecular biology [7, 8]. Compared with classical molecular techniques, RT-qPCR has major advantages of higher sensitivity, better reproducibility and specificity; moreover, with its high-throughput nature, it has become the “gold standard” for gene expression quantification [9]. Nonetheless, the results of RT-qPCR vary due to differences in initial sample size, template RNA integrity, mRNA recovery, reverse transcription efficiency, and primer design. Thus, to obtain accurate and reliable gene expression results, RT-qPCR data must be normalized with appropriate reference genes to make the results more accurate and standardized, and the expression of which should not fluctuate during treatment [2, 10–12]. β-Actin (ACTB), elongation factor 1 (EF1), glyceraldehyde-3-phosphate dehydrogenase (GAPDH), β-tubulin (TUB), and 18S ribosomal RNA (18S rRNA) have been widely used as reference genes in different organisms because they are considered to have uniform expression [2, 12–15]. In addition, although ACTB and GAPDH are the most commonly used reference genes, they have not been validated [16]. Furthermore, studies on reference gene expression in insects have found that the traditional genes show low stability across various biotic and abiotic conditions [16], and the most frequently used reference genes might not be stably expressed under different experimental conditions, causing a high potential for result misinterpretation. Therefore, it is necessary to perform systematic validation of reference genes to maximize the accuracy of PCR analysis and the reliability of published results [17, 18]. Ribosomal protein genes are considered good reference genes because they are expressed in all cell types for the synthesis of new ribosomes [19–21]. Indeed, ribosomal protein genes (ribosomal protein L genes and S genes) exhibit high stability in different tissues of insects [22–29]. Our next study will investigate the gene expression of different tissues in *E*. *scrobiculatus*; therefore, the screening of reference genes is necessary in different tissues.

In this study, we report quantitative analyses of the expression of 18 candidate reference genes in various tissues of *E*. *scrobiculatus*. The 18 genes evaluated were β-actin, RPS3, AK, GAPDH, ribosomal protein S11 (RPS11), RPL18, actin-5C, ACTIN, RPL13, RPL27, β-TUB, α-TUB, eukaryotic translation initiation factor 5 (EIF5), RPL10a, elongation factor 1-alpha (EF1-α), UBC, RPL36, RPL14, and arginine kinase (AK). This study provides a reliable reference for the selection of reference genes for insect gene expression studies or molecular biology.

## 2.Materials and methods

### 2.1. Insects

Adults of *E*. *scrobiculatus* were collected from *A*. *altissima* individuals in Wutongshu Town, Lingwu City, Ningxia Province and kept in the laboratory at 25±1°C for 24 hours under 70 ± 5% humidity and a 16:8-h light/dark (L:D) photoperiod.

### 2.2. Sample preparation

The stability of candidate genes in *E*. *scrobiculatus* was assessed in different tissues of adult males and females. The various tissues examined (10 tissues) included the Malpighian tubule, hindgut, antenna, head, foregut, leg, male genitalia, female genitalia, wing and midgut. Each tissue sample was collected from the same 20 individuals. All samples were frozen at -80°C until RNA extraction was performed.

### 2.3. RNA extraction and cDNA synthesis

Total RNA was extracted from samples using TRIzol (Aidlab Biotechnologies, China) following the manufacturer's instructions. The RNA concentration was quantified by measuring absorbance at 260 nm using a spectrophotometer (NanoDrop 8000, Thermo Fisher Scientific, USA). RNA purity was assessed at absorbance ratios of OD260/280 and OD260/230, and its integrity was determined by 1% agarose gel electrophoresis. Single-stranded cDNA was synthesized using 1 μg of RNA from various samples with a reverse transcription system (Takara, Japan) according to the manufacturer's recommendations. The cDNA was stored at -20°C until further use.

### 2.4. Verification of candidate reference genes

Candidate reference genes evaluated in this study were selected from the *E*. *scrobiculatus* RNA-seq transcriptome dataset (SRP136832) deposited in the NCBI database (https://www.ncbi.nlm.nih.gov/). First, open reading frames (ORFs) of each candidate reference gene sequence were identified by ORF Finder (http://www.ncbi.nlm.nih.gov/gorf/gorf.html), and the full-length ORFs of candidate reference genes (β-actin, RPS3, AK, GAPDH, RPS11, RPL18, actin-5C, ACTIN, RPL13, RPL27, β-TUB, α-TUB, EIF5, RPL10a, EF1-α, UBC, RPL36, RPL14, and AK) were cloned. The conditions for PCR amplification consisted of 95°C for 5 min, 35 cycles of 95°C for 30 s, 57°C for 30 s, and 72°C for 30 s, and 72°C for 5 min. The PCR products were cloned into a pTOPO-Blunt vector (Aidlab Biotech, Beijing, China) and sequenced. The primer sequences used for full-length cloning of candidate reference genes are shown in S1 Table.

### 2.5. Candidate reference genes and primer design

Primers for subsequent RT-qPCR of the selected candidate reference genes were designed using Primer3Plus software (Table 1). Reactions were performed using StepOnePlus (Applied Biosystems). cDNA was amplified using 2× TB Green^™^ Premix Ex Taq^™^ II (TaKaRa, Japan) according to the manufacturer's protocol in final reaction volumes of 20 μL. Each reaction contained 2 μL cDNA as a template, 0.8 μL forward and reverse primers (10 μM), 10 0078L 2× TB Green Premix, 0.4 μL ROX dye, and 7 μL ddH2O. PCR involved 95°C for 1 min and 40 cycles of 95°C for 10 s, 57°C for 10 s and 72°C for 20 s, followed by melting curve analysis (from 60 to 95°C) to ensure the specificity of the amplified product. A dissociation curve analysis was performed for each reaction to confirm the specificity of the amplification. RCR products were cloned and sequenced to confirm the amplification of the correct products. Relative standard curves for the transcripts were generated using serial dilutions of cDNA (1/5, 1/25, 1/125, 1/625, and 1/3125), and the corresponding RT-qPCR efficiencies (E) were calculated using the following equation: E = (10[−1/slope] − 1) × 100. Reactions for three technical replicates were performed independently.

**Table 1:** Primers used for studying reference gene expression in *E*. *scrobiculatus* by RT-qPCR.

Gene	Primer (5’-3’)	Product length	Tm	E	R^2^
β-actin	GAAGGACTTGTACGCCAACACTGT	185	59.2	89.79	0.99915
	AGGTGGAGAGGGAAGCCAAGATG		60.1		
RPL18	GTGGCGAAATCCCGTTTCTGGTA	151	59.6	85.85	0.99905
	CGGAACGAGAGTCATACCTTAGCC		59.1		
RPS3	TCAACAAGTATCCATCGGAAGTCT	220	56.2	94.94	0.99804
	AATGTCCTTGGCGATACTGTCA		55.8		
GAPDH	TCTCCAACGCCTCTTGTACTACCA	200	59.6	87.55	0.99967
	AGCACCAGTGGACGCAGGAA		59.7		
AK	ATGGTTGACGCCGCAGTTCTC	212	59.5	93.89	0.99565
	GCATCAGGAGCATAGATACCGATACC		59.5		
RPS11	GCAAGTAATCACGGCGGATAACG	190	59.5	92.74	0.99967
	AAGAAGGTGGTCAGGTTGTCCAGAA		59.3		
actin-5C	CGCCATTCTCCGTCTGGACTTG	100	59.6	100.19	0.99809
	TCGCTCAGCAGTGGTGGTGAA		60.1		
ACTIN	GTCAGGTCATCACCATCGGTAACG	160	59.8	95.26	0.99983
	ACAGTGTTGGCGTACAAGTCCTTC		59.8		
RPL13	ACAGAAGGAAGCGTGTTGTGGTT	106	59.3	96.09	0.99181
	TTCCAGCCAGCCTCGTGTGA		59.5		
RPL27	ACATGCTTTGGTCGCTGGTATCG	192	60.0	95.52	0.99821
	GGGTCTTTGAGGTCTTTACTGGTGAC		60.0		
β-TUB	CGGCTACCTTCATCGGCAACTC	199	59.7	94.59	0.99734
	GCGGTGGCTTCCTGGTATTGTT		59.5		
α-TUB	GTTGGAGGTGGAGACGACAGTT	202	58.3	88.42	0.99735
	TTCCTTGCCGATGGTGTAGTGG		58.6		
EIF5	GCCTCCTTATCAGCAACCACAAACTA	162	60.0	95.79	0.99605
	CCATACCAGACTCATCGGCATTATCC		59.8		
RPL10a	TCTTGGCGATCAGCAGCATTGTG	225	60.4	92.27	0.99849
	TCTTGATGAGACAGCAGACCTGGAA		60.1		
EF1-α	AGGCTGACTGTGCTGTTCTTATTGT	171	59.4	87.97	0.99446
	CTTCGCTGTATGGTGGTTCAGTAGAG		59.9		
UBC	CTCTCGAAGTAGAGCCTTCTGATACA	187	58.3	94.57	0.99915
	CCACCTCTCAACCTCAACACCAA		58.8		
RPL36	CCCTGACGAACTTGGTGTGATGG	131	59.8	96.74	0.99774
	ATACGAGATCGCCGTGGGTCTTAA		60.0		
RPL14	AGGTTGTCCGTAAGGCGTGGAA	145	60.2	94.67	0.99824
	CTGCGAGTTCTAGCTCTCCTGAGT		60.0		

### 2.6. Constancy analysis of candidate reference genes

The Ct value is the number of amplification cycles that are elapsed when the fluorescence signal of the amplified product reaches the set threshold during PCR amplification. Data analyses were analyzed independently for each adult tissue of Ct values. The stability of the reference gene means that the expression levels should be approximate and no significant differences were observed under various types of tissues and various experimental conditions. The stability of the 18 candidate reference genes was evaluated using geNorm, NormFinder, and BestKeeper as well as the delta Ct method in Microsoft Excel.

The geNorm algorithm determines an expression stability value (M) for each gene and then compares the pairwise variation (V) in this candidate reference gene with that in other tested candidate reference genes. Candidate reference genes with lower M-values have more stable expression. If the pairwise variation (V_n_/V_n+1_) between sequential normalization factors is less than 0.15, then it is necessary to use n genes as reference genes for that particular experimental condition [30]. NormFinder uses a model-based method to estimate the variation in expression of candidate reference genes, assigning a stability value to each candidate reference gene, whereby candidate reference genes with lower values are more stable [31]. BestKeeper calculates the standard deviation (SD) and stability value (SV) of candidate reference genes based on raw data (CT values), and those with low index scores are considered to be highly stable [32]. The delta Ct method calculates the mean SD by pairwise comparisons; a lower SD indicates a more stable gene [33]. Finally, RefFinder, a web-based comprehensive algorithm used to evaluate and screen candidate reference genes, integrates four computational programs (geNorm, NormFinder, BestKeeper, and delta Ct) to rank candidate reference genes. RefFinder is a comprehensive program that ranks candidate reference genes based on the rankings from four algorithms; each candidate reference gene is assigned an appropriate weight to determine a comprehensive final ranking (http://150.216.56.64/referencegene.php?type=reference).

### 2.7. Validation of a selected reference gene

To further confirm the reliability of candidate reference genes, the relative expression profiles of GH45 were determined in ten tissues and normalized to the two most stable candidate reference genes (RPL13 and RPS3) and the least stable gene (actin-5C). The relative quantity of the target gene was calculated using the 2^−ΔΔCt^ method [34]. To determine the effect on the relative expression of the target gene GH45 when different reference genes were used, we performed an independent sample t test. Primers used for amplifying and quantifying GH45 in *E*. *scrobiculatus* are provided in S2 Table.

## 3. Results

### 3.1. PCR amplification of candidate reference genes

Based on the results of the dissociation curves, a single peak but no signal in the negative controls for each reaction were obtained, suggesting that each gene was specifically amplified. Candidate reference gene sequences were amplified correctly by gel electrophoresis, and all the fragments were cloned and sequenced. The standard curves of candidate genes revealed correlation coefficient (R^2^) values for all primer pairs ranging from 0.99605 to 0.99983, and the PCR efficiency determined by the standard curve ranged from 85.85 to 100.19% (S1 Fig, Table 1).

### 3.2. Expression profiling of candidate reference genes in *E*. *scrobiculatus*

**Fig 1:**
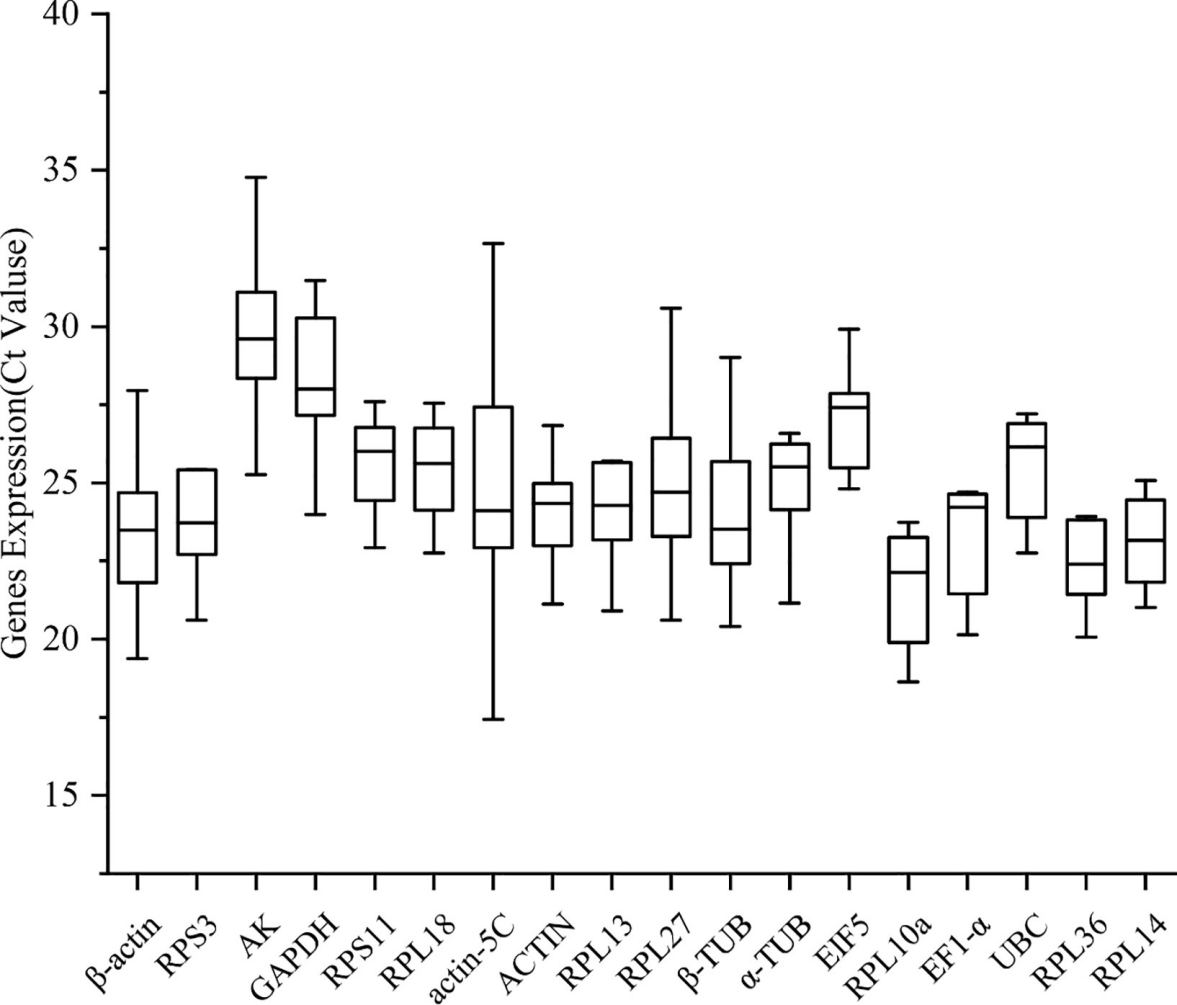
Expression profiles of 18 candidate reference genes based on different tissue.

To obtain an overview of transcript abundance, expression levels of the 18 candidate reference genes were investigated in different tissues of *E*. *scrobiculatus*. The variable Ct value of all candidate reference genes across different tissues showed different ranges of expression and different expression patterns for the 18 genes across various experimental groups (Fig 1). The Ct values for the 18 genes were used to estimate the stability of gene expression among the tissue samples (Table 1). The mean Ct values of the candidate reference genes ranged from 22.49 for ribosomal protein L10a (RPL10a) to 29.97 for arginine kinase (AK), which exhibited the most and least abundant transcripts, respectively (Fig 1).

### 3.3. Expression stability and ranking of candidate reference genes

**Fig 2:**
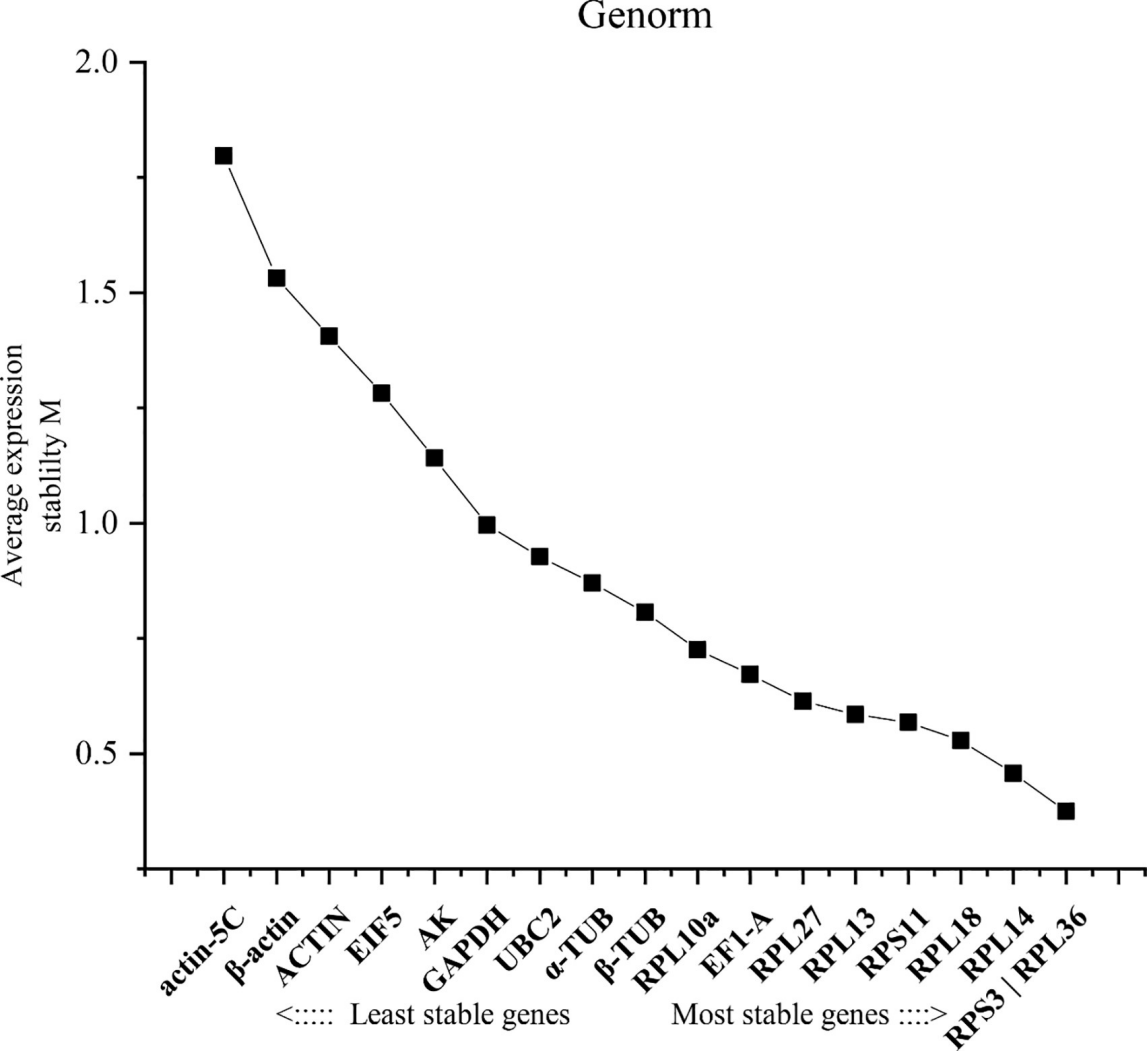
Expression stability and relative ranking of the 18 reference genes predicted by geNorm. The mean expression stability (M) was calculated by stepwise exclusion of the least stable gene across all the tissues within a particular group set. The mean stability of different genes is plotted; the least stable genes are represented on the left and the most stable on the right side of the plot.

**Fig 3:**
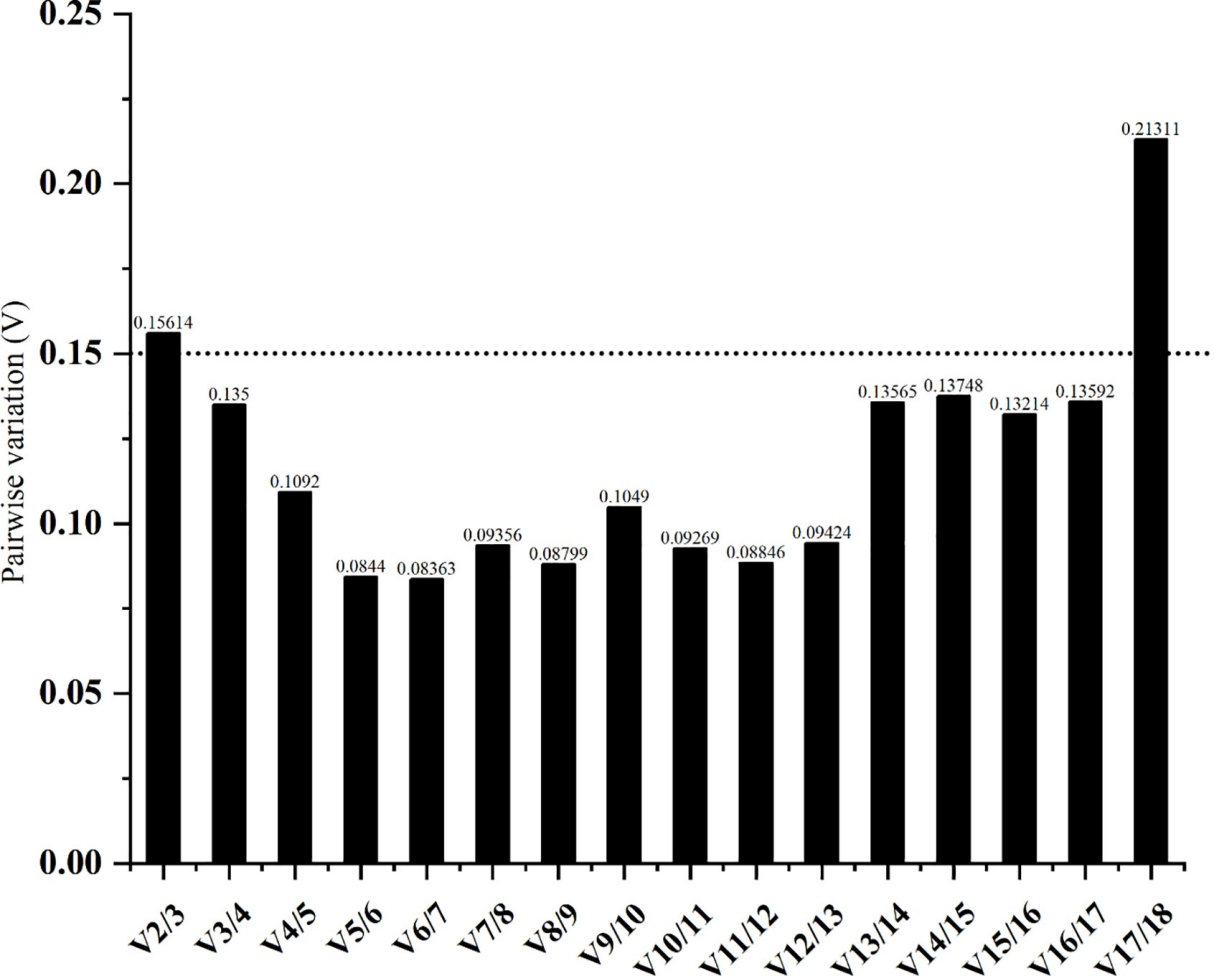
Pairwise variation (V) values using geNorm based on different tissues. Average pairwise variations (V) were calculated between the normalization factors NFn and NFn + 1 by geNorm software to indicate the optimum number of reference genes required for RT-qPCR data normalization. A threshold value below 0.15 indicated that the additional reference gene has no significant improvement on normalization in RT-qPCR data.

Four statistical algorithms were used to rank the candidate reference genes based on expression stability across different tissues. From the **geNorm** analysis, the pairwise variation value of V2/3 was less than the cut-off value of 0.15 (Fig 2). Thus RPS3, RPL36 and RPL14 were ranked the most stable genes based on **geNorm** tissue-specific expression profiling (Fig 3). According to the rank order assigned by **NormFinder**, RPL13, RPL27 and RPS3 were the suitable genes for tissue-specific experiments (see Table 2). **BestKeeper** considered ACTIN, RPL13 and RPS11 to show the most suitable expression, which was rated as the best reference gene (Table 2). Lastly, the **delta Ct** method selected RPL13, RPS3 and RPL27 as the most suitable reference gene among the various tissues (Table 2).

**Table 2:** Reference gene expression stability of different tissues based on five algorithms.

Reference gene	geNorm		NormFider		BestKeeper		delta Ct		RefFinder	
	Stability	Rank	Stability	Rank	Stability	Rank	Stability	Rank	Stability	Rank
β-actin	1.532	16	2.060	16	2.478	17	2.440	16	16.49	17
RPS3	0.375	1	0.501	3	1.968	10	1.289	2	2.78	2
AK	1.141	13	1.469	14	2.184	13	2.047	14	13.74	15
GAPDH	0.996	12	1.037	10	2.371	16	1.703	12	12.57	14
RPS11	0.568	4	0.704	5	1.663	3	1.384	6	4.61	5
RPL18	0.528	3	0.693	4	1.845	6	1.368	4	4.43	4
actin-5C	1.797	17	3.825	18	3.121	18	3.920	18	18.00	18
ACTIN	1.406	15	1.753	15	1.648	1	2.224	15	7.75	9
RPL13	0.585	5	0.358	1	1.659	2	1.277	1	1.86	1
RPL27	0.613	6	0.497	2	1.986	11	1.336	3	4.64	6
β-TUB	0.807	9	0.705	6	1.899	9	1.516	8	8.11	10
α-TUB	0.870	10	0.871	8	1.689	4	1.567	10	7.50	8
EIF5	1.282	14	2.256	17	2.314	15	2.523	17	15.97	16
RPL10a	0.725	8	1.247	12	2.220	14	1.632	11	11.36	12
EF1-α	0.672	7	1.072	11	1.894	8	1.572	9	9.16	11
UBC	0.927	11	1.336	13	2.101	12	1.731	13	12.49	13
RPL36	0.375	1	0.779	7	1.851	7	1.373	5	3.96	3
RPL14	0.457	2	0.986	9	1.827	5	1.452	7	5.54	7

### 3.4. Comprehensive ranking of candidate reference genes

**Fig 4:**
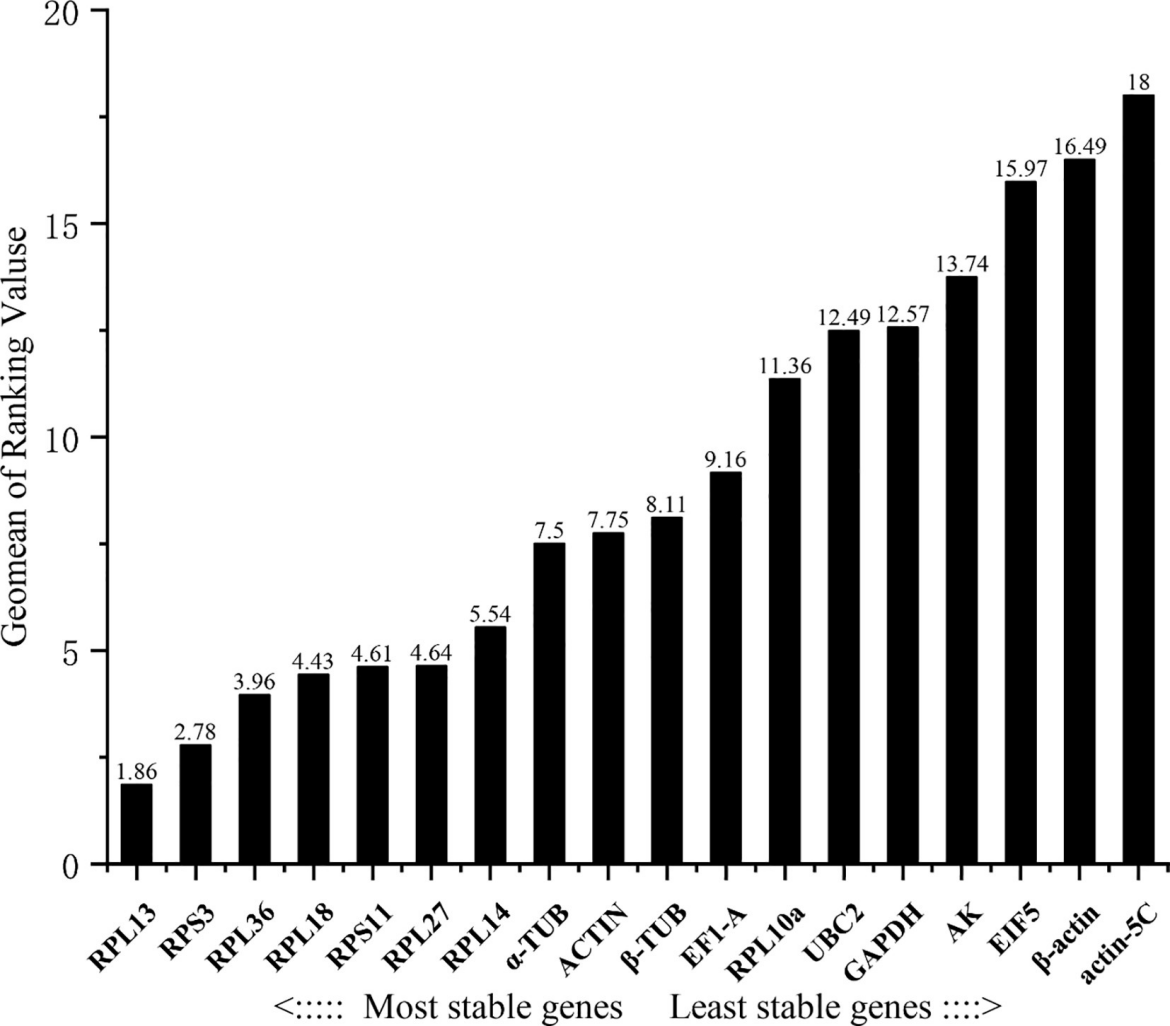
Stability of candidate reference genes expression under different tissues. A lower Geomean value indicates more stable expression based on RefFinder.

Four algorithms ranked the candidate reference genes separately, and then RefFinder was used for comprehensive ranking. The following rankings given by RefFinder are listed in decreasing order of stability of tissue-related expression: RPL13, RPS3, RPL36, RPL18, RPS11, RPL27, RPL14, α-TUB, ACTIN, β-TUB, EF1-A, RPL10a UBC, GAPDH, AK, EIF5, β-actin, and actin-5C (Fig 4).

### 3.5. Validation of the recommended candidate reference genes

**Fig 5:**
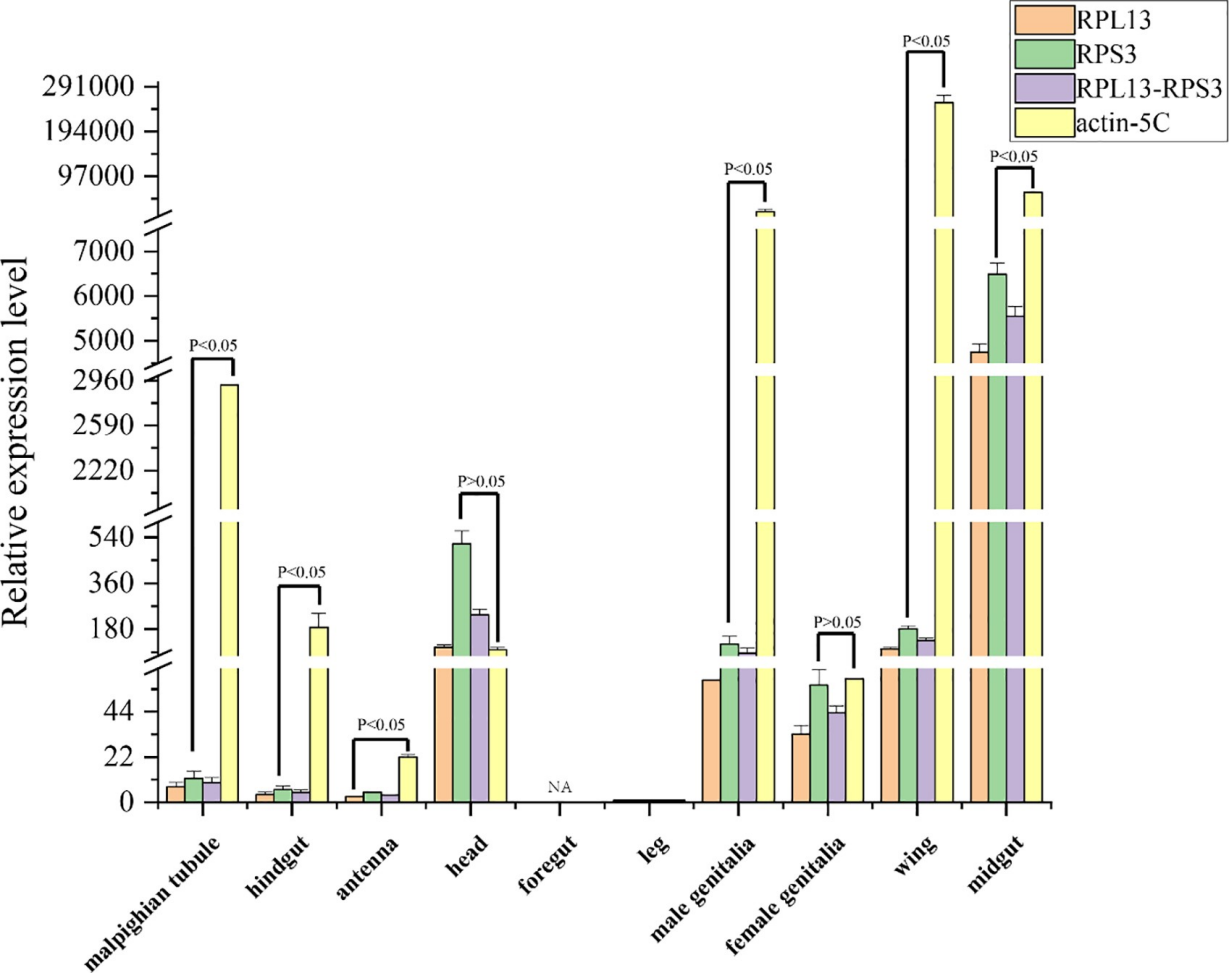
Validation of the candidate reference genes. Expression profiles of GH45 were investigated using different tissues. The expression of GH45 was normalized using the best reference gene (RPL13), the second best reference gene (RPS3), the top two NF (RPL13–RPS3) and the worst reference gene (actin-5C). Bars represent the means and standard deviation of three technical replications. Bars represent the means and standard deviations of three technical replicates.

In our study, the comprehensive rankings from RefFinder showed that RPL14 and RPS3 had the greatest stability in various tissues, whereas actin-5C showed the lowest stability. The normalization applicability of these candidate reference genes alone and in combination was tested to obtain expression profiles for GH45. Similar results were obtained using either one (RPL13 or RPS3) or two (RPL13-RPS3) candidate reference genes for normalization (Fig 5). However, when actin-5C was used as a reference gene to standardize GH45, GH45 gene expression showed a huge difference in standardization with two other genes as references. Similar expression levels of GH45 were observed when normalized using RPL13, RPS3 and RPL13-RPS3 in different tissues. However, the expression levels of GH45 normalized using actin-5C and other candidate reference genes were significantly different in those tissues (P<0.05, Fig 5). These results indicated that the expression levels of genes are significantly different when unstable reference genes are used to normalized qRT-PCR data in different tissues. The results further verified that appropriate selection of candidate reference genes is necessary for the investigation of gene expression levels.

## 4. Discussion

Many biological processes are closely related to the expression level of genes, and detection of gene expression levels is very important. Due to its high accuracy, sensitivity, specificity, and rapidity, RT-qPCR has been widely used for validating the results of gene expression profiles [35]. However, RT-qPCR requires normalization by reference genes to offset variation among samples processed from different tissues [36]. In recent years, with the gradual deepening of insect molecular biology research, it is becoming increasingly necessary to select appropriate reference genes to evaluate many biological processes or important functional genes in insects [2, 3, 22, 24, 29, 37–47]. Regardless, there is no study to date on reference genes in *E*. *scrobiculatus*. Herein, we attempt to identify the most suitable reference genes for RT-qPCR analyses of samples from different tissues of this pest.

Comparative analyses revealed some differences among the different statistical methods with regard to the rankings of the stability of the 18 candidate reference genes (Table 2). The geNorm algorithm calculates the pairwise variation (V_n_ / V_n+1_) between continuous standardization factors to determine the optimal number of reference genes [30]. NormFinder analyzes expression variation in reference genes to estimate their stability [31], and BestKeeper uses pairwise correlations to assess stability among reference genes [48]. The delta Ct method analyzes the relative expression of gene pairs to identify suitable reference genes [33]. In our study, the delta Ct method results were similar to those of geNorm and NormFinder, and all stable reference genes were ribosomal protein genes, which in previous studies were identified as being highly stable between different insect tissues [35]. However, the rankings by BestKeeper differed greatly from those of the other three algorithms, similar to the results of previous reports [44, 49]. We also utilized the web-based comprehensive tool RefFinder, which integrates the four algorithms, to rank the overall stability of the 18 reference genes. Overall, our results suggest that it is necessary to use different statistical methods to assess the stability of reference genes in different tissues.

β-Actin and GAPDH are usually selected as reference genes for RT-qPCR, but under many conditions, these two genes are not always ideal or even suitable reference genes [16]. Therefore, we selected three genes, namely, ACTIN, β-actin, and actin-5C, as candidate reference genes. However, ACTIN, β-actin, and actin-5C were mainly the most unstable candidate reference genes according to our results, and the stability of GAPDH was also low. In contrast, RPL13 showed the most stable expression in different tissues. Recent studies of potential reference genes in insects have found that ribosomal protein genes are among the most stable, especially between different tissues [35]. This result is consistent with that of reference gene analysis in *Solenopsis invicta* (Hymenoptera: Formicidae) [28] and *Bemisia abaci* (Hemiptera: Aleyrodidae) [29]. TUB belongs to the small globular family of proteins and is the major building block of microtubules in most eukaryotic cells. α-TUB and β-TUB were used in our study, and their rankings were very similar among the four methods. AK, a type of phosphagen kinase that is widely distributed in various tissues of invertebrates, exhibits high stability between different tissues of *Drosophila suzukii* and *Bombus lucorum* [50, 51]. Eukaryotic translation initiation factor (EIF) is involved in the initiation of eukaryotic translation and is the most stable gene among different tissues of *Bombyx mori* [52]. UBC and EF1-α are also commonly used as reference genes [22, 35], but in our study, these genes were not as stable as were ribosomal protein genes. Ribosomal protein genes were found to be the most stable reference genes in different tissues in *E*. *scrobiculatus*, consistent with the results of a previous study [35].

Glycoside hydrolase family 45 is a known insect endogenous cellulolytic family [53, 54], and in this study, reference genes were validated by determining the expression profiles of the target gene GH45. As the expression level of GH45 was significantly different when normalization was based on RPL13, RPS3 and two reference genes (RPL13-RPS3) and on actin-5C, the normalization results based on actin-5C did not accurately reflect the expression level of GH45. These results are necessary for the selection of reference genes, suggesting that it is important to standardize the expression of target genes in *E*. *scrobiculatus* using a stable reference gene.

Overall, this is the first study to evaluate candidate reference genes for gene expression analyses in *E*. *scrobiculatus*. The expression profiles of 18 candidate reference genes in different tissues were investigated using four algorithms and RefFinder. We further confirmed that the reference genes commonly used may not be suitable for RT-qPCR normalization; consequently, reference genes must be verified before use. Nevertheless, we hereby recommend that RPL13 is the most stable reference gene for various tissues. The findings of this study not only provide stable reference genes for quantification of gene expression in *E*. *scrobiculatus* but also lay the foundation for transcriptomics and functional gene research on this pest.

## Supporting information

S1 Fig Standard curves of the 18 candidate reference genes. (DOCX)

S2 Fig Candidate reference gene selection procedure. (DOCX)

S3 Fig Agarose gel electrophoresis of RPC amplification products of ten candidate reference genes. (DOCX)

S1 Table Standard curves of the 18 candidate reference genes. (DOCX)

S2 Table Primers used for amplifying and quantified GH45 in *E*. *scrobiculatus*. (DOCX)

S3 Table Average Ct values of 18 candidate reference genes. (DOCX)

S4 Table Stability values of 18 candidate reference genes given by four algorithms. (DOCX)

## References

[1] Ding J, Reardon R, Wu Y, Zheng H, Fu W. Biological control of invasive plants through collaboration between China and the United States of America: a perspective. Biological Invasions. 2006;8(7):1439–50. 10.1007/s10530-005-5833-2

[2] Xu Jing, Lu Ming-Xing, Cui Ya-Dong, Du Y-Z. Selection and Evaluation of Reference Genes for Expression Analysis Using qRT-PCR in Chilo suppressalis (Lepidoptera_ Pyralidae). Journal of Economic Entomology. 2017 10.1093/jee/tow297 28115499

[3] Tan Y, Zhou XR, Pang BP. Reference gene selection and evaluation for expression analysis using qRT-PCR in Galeruca daurica (Joannis). Bull Entomol Res. 2017;107(3):359–68. Epub 2016/11/08. 10.1017/S0007485316000948 . 27819206

[4] Ji Y, Luo W, Zhang G, Wen J. Projecting potential distribution of Eucryptorrhynchus scrobiculatus Motschulsky and E. brandti (Harold) under historical climate and RCP 8.5 scenario. Sci Rep. 2017;7(1):9163 10.1038/s41598-017-09659-3 28831145 PMC5567332

[5] Liu ZK, Wen JB. Transcriptomic Analysis of Eucryptorrhynchus chinensis (Coleoptera: Curculionidae) Using 454 Pyrosequencing Technology. J Insect Sci. 2016;16(1). 10.1093/jisesa/iew067 27620556 PMC5019023

[6] Ding J, WU Y, ZHENG H, FU W, REARDON R, LIU M. Assessing potential biological control of the invasive plant, tree-of-heaven, Ailanthus altissima. Biocontrol Science and Technology. 2006 10.1080/09583150500531909

[7] Huggett J, Dheda K, Bustin S, Zumla A. Real-time RT-PCR normalisation; strategies and considerations. Genes Immun. 2005;6(4):279–84. 10.1038/sj.gene.6364190 . 15815687

[8] Stephen A Bustin, Jean-François Beaulieu, Huggett Jim, Jaggi Rolf, Frederick SB Kibenge, Pål A Olsvik, et al MIQE précis_ Practical implementation of minimum standard guidelines for fluorescencebased quantitative real-time PCR experiments. BMC Molecular Biology. 2010;11(74). 10.1186/1471-2199-11-74 PMC2955025 20858237

[9] Bustin SA, Nolan T. Analysis of mRNA Expression by Real-Time PCR. Real-time PCR: advanced technologies and applications Caister Academic Press 2013;pp(51–88).

[10] Bustin SA, Benes V, Garson J, Hellemans J, Huggett J, Kubista M, et al The need for transparency and good practices in the qPCR literature. Nat Methods. 2013;10(11):1063–7. 10.1038/nmeth.2697 . 24173381

[11] Nicot N, Hausman JF, Hoffmann L, Evers D. Housekeeping gene selection for real-time RT-PCR normalization in potato during biotic and abiotic stress. J Exp Bot. 2005;56(421):2907–14. 10.1093/jxb/eri285 . 16188960

[12] Dheda K, Huggett JF, Bustin SA, Johnson MA, Rook G, A Z. Validation of housekeeping genes for normalizing RNA expression in real-time PCR. Biotechnology. 2004;37(112–119). 10.2144/04371RR03 15283208

[13] de Kok JB, Roelofs RW, Giesendorf BA, Pennings JL, Waas ET, Feuth T, et al Normalization of gene expression measurements in tumor tissues: comparison of 13 endogenous control genes. Lab Invest. 2005;85(1):154–9. 10.1038/labinvest.3700208 . 15543203

[14] Radonić A, Thulke S, Mackay IM, Landt O, Siegert W, Nitsche A. Guideline to reference gene selection for quantitative real-time PCR. Biochemical and Biophysical Research Communications. 2004;313(4):856–62. 10.1016/j.bbrc.2003.11.177 14706621

[15] Ibanez F, Tamborindeguy C. Selection of reference genes for expression analysis in the potato psyllid, Bactericera cockerelli. Insect Mol Biol. 2016;25(3):227–38. 10.1111/imb.12219 . 26936438

[16] Suzuki Toshihide, Higgins Paul J., Crawford DR. Control Selection for RNA Quantitation. BioTechniques. 2000;29:332–7. 10.2144/00292rv02 10948434

[17] Lee PD, Sladek R, Greenwood CM, Hudson TJ. Control genes and variability: absence of ubiquitous reference transcripts in diverse mammalian expression studies. Genome Res. 2002;12(2):292–7. Epub 2002/02/06. 10.1101/gr.217802 11827948 PMC155273

[18] Gutierrez L, Mauriat M, Guenin S, Pelloux J, Lefebvre JF, Louvet R, et al The lack of a systematic validation of reference genes: a serious pitfall undervalued in reverse transcription-polymerase chain reaction (RT-PCR) analysis in plants. Plant Biotechnol J. 2008;6(6):609–18. Epub 2008/04/25. 10.1111/j.1467-7652.2008.00346.x . 18433420

[19] Hsiao LL, Dangond F, Yoshida T, Hong R, Jensen RV, Misra J, et al A compendium of gene expression in normal human tissues. Physiol Genomics. 2001;7(2):97–104. 10.1152/physiolgenomics.00040.2001 . 11773596

[20] de Jonge HJ, Fehrmann RS, de Bont ES, Hofstra RM, Gerbens F, Kamps WA, et al Evidence based selection of housekeeping genes. PLoS One. 2007;2(9):e898 10.1371/journal.pone.0000898 17878933 PMC1976390

[21] Popovici Vlad, Goldstein Darlene R, Antonov Janine, Jaggi Rolf, Delorenzi Mauro, Wirapati P. Selecting control genes for RT-QPCR using public microarray data. BMC Bioinform. 2009; 10.1186/1471-2105-10-42. PMC2640357 19187545

[22] Lu Y, Yuan M, Gao X, Kang T, Zhan S, Wan H, et al Identification and validation of reference genes for gene expression analysis using quantitative PCR in Spodoptera litura (Lepidoptera: Noctuidae). PLoS One. 2013;8(7):e68059 Epub 2013/07/23. 10.1371/journal.pone.0068059 23874494 PMC3706614

[23] Rodrigues TB, Khajuria C, Wang H, Matz N, Cunha Cardoso D, Valicente FH, et al Validation of reference housekeeping genes for gene expression studies in western corn rootworm (Diabrotica virgifera virgifera). PLoS One. 2014;9(10):e109825 10.1371/journal.pone.0109825 25356627 PMC4214676

[24] Miao Yuan, Yanhui Lu, Xun Zhu, Hu Wan, Muhammad Shakeel, Sha Zhan, et al Selection and Evaluation of Potential Reference Genes for Gene Expression Analysis in the Brown Planthopper, Nilaparvata lugens (Hemiptera_ Delphacidae) Using Reverse-Transcription Quantitative PCR. PLoS ONE. 2014;9(e111369). 10.1371/journal PMC3900570 24466124

[25] Zhang S, An S, Li Z, Wu F, Yang Q, Liu Y, et al Identification and validation of reference genes for normalization of gene expression analysis using qRT-PCR in Helicoverpa armigera (Lepidoptera: Noctuidae). Gene. 2015;555(2):393–402. 10.1016/j.gene.2014.11.038 . 25447918

[26] Sun M, Lu MX, Tang XT, Du YZ. Exploring valid reference genes for quantitative real-time PCR analysis in Sesamia inferens (Lepidoptera: Noctuidae). PLoS One. 2015;10(1):e0115979 10.1371/journal.pone.0115979 25585250 PMC4293147

[27] Lu Y, Zheng X, Liang Q, Xu H, Yang Y, Tian J, et al Evaluation and validation of reference genes for SYBR Green qRT-PCR normalization in Sesamia inferens (Lepidoptera: Noctuidae). Journal of Asia-Pacific Entomology. 2015;18(4):669–75. 10.1016/j.aspen.2015.08.002

[28] Cheng D, Zhang Z, He X, Liang G. Validation of reference genes in Solenopsis invicta in different developmental stages, castes and tissues. PLoS One. 2013;8(2):e57718 Epub 2013/03/08. 10.1371/journal.pone.0057718 23469057 PMC3585193

[29] Li R, Xie W, Wang S, Wu Q, Yang N, Yang X, et al Reference gene selection for qRT-PCR analysis in the sweetpotato whitefly, Bemisia tabaci (Hemiptera: Aleyrodidae). PLoS One. 2013;8(1):e53006 Epub 2013/01/12. 10.1371/journal.pone.0053006 23308130 PMC3540095

[30] Vandesompele J, De Preter K, Pattyn F, Poppe B, Van Roy N, De Paepe A, et al Accurate normalization of real-time quantitative RT-PCR data by geometric averaging of multiple internal control genes. Genome Biology. 2002;3(7). 10.1186/gb-2002-3-7-research0034 PMC126239 12184808

[31] Andersen CL, Jensen JL, Orntoft TF. Normalization of real-time quantitative reverse transcription-PCR data: a model-based variance estimation approach to identify genes suited for normalization, applied to bladder and colon cancer data sets. Cancer Res. 2004;64(15):5245–50. Epub 2004/08/04. 10.1158/0008-5472.CAN-04-0496 . 15289330

[32] Pfaffl Michael W., Tichopad Ales, Prgomet Christian, Neuvians TP. Determination of stable housekeeping genes, differentially regulated target genes and sample integrity: BestKeeper–Excel-based tool using pair-wise correlations. Biotechnology Letters. 2004;26:509–515. 10.1023/b:bile.0000019559.84305.47 15127793

[33] Silver N, Best S, Jiang J, Thein SL. Selection of housekeeping genes for gene expression studies in human reticulocytes using real-time PCR. BMC Mol Biol. 2006;7:33 Epub 2006/10/10. 10.1186/1471-2199-7-33 17026756 PMC1609175

[34] Livak KJ, Schmittgen TD. Analysis of relative gene expression data using real-time quantitative PCR and the 2(-Delta Delta C(T)) Method. Methods. 2001;25(4):402–8. Epub 2002/02/16. 10.1006/meth.2001.1262 . 11846609

[35] Shakeel M, Rodriguez A, Tahir UB, Jin F. Gene expression studies of reference genes for quantitative real-time PCR: an overview in insects. Biotechnol Lett. 2018;40(2):227–36. 10.1007/s10529-017-2465-4 . 29124515

[36] Yang C, Pan H, Liu Y, Zhou X. Stably Expressed Housekeeping Genes across Developmental Stages in the Two-Spotted Spider Mite, Tetranychus urticae. Plos One. 2015;10(3). 10.1371/journal.pone.0120833 25822495 PMC4379063

[37] Fu W, Xie W, Zhang Z, Wang S, Wu Q, Liu Y, et al Exploring valid reference genes for quantitative real-time PCR analysis in Plutella xylostella (Lepidoptera: Plutellidae). Int J Biol Sci. 2013;9(8):792–802. Epub 2013/08/29. 10.7150/ijbs.5862 23983612 PMC3753443

[38] Sinha DK, Smith CM. Selection of reference genes for expression analysis in Diuraphis noxia (Hemiptera: Aphididae) fed on resistant and susceptible wheat plants. Sci Rep. 2014;4:5059 Epub 2014/05/28. 10.1038/srep05059 24862828 PMC4034006

[39] Koramutla MK, Aminedi R, Bhattacharya R. Comprehensive evaluation of candidate reference genes for qRT-PCR studies of gene expression in mustard aphid, Lipaphis erysimi (Kalt). Sci Rep. 2016;6:25883 Epub 2016/05/12. 10.1038/srep25883 27165720 PMC4863174

[40] Zhang QL, Zhu QH, Liao X, Wang XQ, Chen T, Xu HT, et al Selection of reliable reference genes for normalization of quantitative RT-PCR from different developmental stages and tissues in amphioxus. Sci Rep. 2016;6:37549 Epub 2016/11/22. 10.1038/srep37549 27869224 PMC5116582

[41] Zhu X, Yuan M, Shakeel M, Zhang Y, Wang S, Wang X, et al Selection and evaluation of reference genes for expression analysis using qRT-PCR in the beet armyworm Spodoptera exigua (Hubner) (Lepidoptera: Noctuidae). PLoS One. 2014;9(1):e84730 Epub 2014/01/24. 10.1371/journal.pone.0084730 24454743 PMC3893131

[42] Yang C, Pan H, Liu Y, Zhou X. Selection of reference genes for expression analysis using quantitative real-time PCR in the pea aphid, Acyrthosiphon pisum (Harris) (Hemiptera, Aphidiae). PLoS One. 2014;9(11):e110454 Epub 2014/11/26. 10.1371/journal.pone.0110454 25423476 PMC4244036

[43] Shen Guang-Mao, Jiang Hong-Bo, Wang Xiao-Na, Wang J-J. Evaluation of endogenous references for gene expression profiling in different tissues of the oriental fruit fly Bactrocera dorsalis (Diptera_ Tephritidae). BMC Molecular Biology. 2010;11:76. 20923571 10.1186/1471-2199-11-76 PMC2972281

[44] Gao XK, Zhang S, Luo JY, Wang CY, Lu LM, Zhang LJ, et al Comprehensive evaluation of candidate reference genes for gene expression studies in Lysiphlebia japonica (Hymenoptera: Aphidiidae) using RT-qPCR. Gene. 2017;637:211–8. Epub 2017/10/02. 10.1016/j.gene.2017.09.057 . 28964897

[45] Qu C, Wang R, Che W, Zhu X, Li F, Luo C. Selection and evaluation of reference genes for expression analysis using quantitative real-time PCR in the Asian Ladybird Harmonia axyridis (Coleoptera: Coccinellidae). PLoS One. 2018;13(6):e0192521 Epub 2018/06/12. 10.1371/journal.pone.0192521 29889877 PMC5995347

[46] Dzaki N, Ramli KN, Azlan A, Ishak IH, Azzam G. Evaluation of reference genes at different developmental stages for quantitative real-time PCR in Aedes aegypti. Sci Rep. 2017;7:43618 Epub 2017/03/17. 10.1038/srep43618 28300076 PMC5353741

[47] Kang ZW, Liu FH, Tian HG, Zhang M, Guo SS, Liu TX. Evaluation of the reference genes for expression analysis using quantitative real-time polymerase chain reaction in the green peach aphid, Myzus persicae. Insect Sci. 2017;24(2):222–34. Epub 2016/01/11. 10.1111/1744-7917.12310 . 26749166

[48] Pfaffl Michael W., Tichopad Ales, Prgomet Christian, Neuvians TP. Determination of stable housekeeping genes, differentially regulated target genes and sample integrity_ BestKeeper–Excel-based tool using pair-wise correlations. Biotechnology Letters. 2004;26:509–515. 10.1023/b:bile.0000019559.84305.47 15127793

[49] Petriccione M, Mastrobuoni F, Zampella L, Scortichini M. Reference gene selection for normalization of RT-qPCR gene expression data from Actinidia deliciosa leaves infected with Pseudomonas syringae pv. actinidiae. Sci Rep. 2015;5:16961 Epub 2015/11/20. 10.1038/srep16961 26581656 PMC4652207

[50] Hornakova D, Matouskova P, Kindl J, Valterova I, Pichova I. Selection of reference genes for real-time polymerase chain reaction analysis in tissues from Bombus terrestris and Bombus lucorum of different ages. Anal Biochem. 2010;397(1):118–20. Epub 2009/09/16. 10.1016/j.ab.2009.09.019 . 19751695

[51] Zhai Y, Lin Q, Zhou X, Zhang X, Liu T, Yu Y. Identification and validation of reference genes for quantitative real-time PCR in Drosophila suzukii (Diptera: Drosophilidae). PLoS One. 2014;9(9):e106800 Epub 2014/09/10. 10.1371/journal.pone.0106800 25198611 PMC4157791

[52] Wang G, Chen Y, Zhang X, Bai B, Yan H, Qin D, et al Selection of reference genes for tissue/organ samples on day 3 fifth-instar larvae in silkworm, Bombyx mori. Arch Insect Biochem Physiol. 2018;98(2):e21458 Epub 2018/03/24. 10.1002/arch.21458 . 29570841

[53] Busch A, Kunert G, Wielsch N, Pauchet Y. Cellulose degradation in Gastrophysa viridula (Coleoptera: Chrysomelidae): functional characterization of two CAZymes belonging to glycoside hydrolase family 45 reveals a novel enzymatic activity. Insect Mol Biol. 2018;27(5):633–50. Epub 2018/05/19. 10.1111/imb.12500 . 29774620

[54] Fischer R, Ostafe R, Twyman RM. Cellulases from insects. Adv Biochem Eng Biotechnol. 2013;136:51–64. Epub 2013/06/04. 10.1007/10_2013_206 . 23728162

