# Supplementary figures and images for: Selection of reference genes for tissue/organ samples of adults of Eucryptorrhynchus scrobiculatus
Source: PLoS One. 2020 Feb 3;15(2):e0228308. doi: 10.1371/journal.pone.0228308 (PMC6996836; doi:10.1371/journal.pone.0228308)

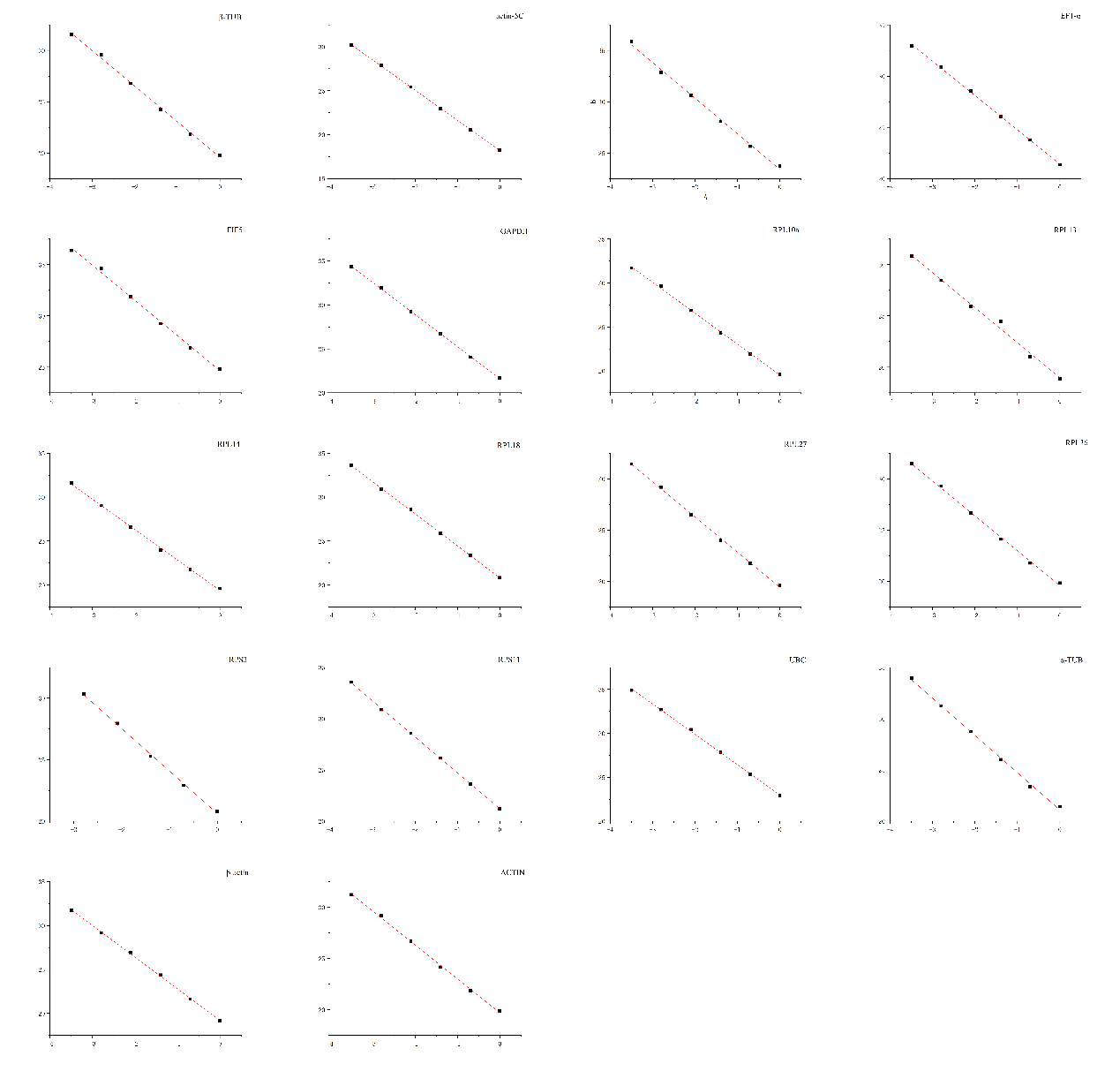


**Fig S1. Standard curves of the 18 candidate reference genes.**

Supplement: S1 Fig — (DOCX) [file pone.0228308.s001.docx]
