# Supplementary material for: Selection of reference genes for tissue/organ samples of adults of Eucryptorrhynchus scrobiculatus
Source: PLoS One. 2020 Feb 3;15(2):e0228308. doi: 10.1371/journal.pone.0228308 (PMC6996836; doi:10.1371/journal.pone.0228308)
Supplement: S2 Fig — (DOCX) [file pone.0228308.s002.docx]

**

**

**Fig S2. Candidate reference gene selection procedure.**
