# Supplementary material for: Selection of reference genes for tissue/organ samples of adults of Eucryptorrhynchus scrobiculatus
Source: PLoS One. 2020 Feb 3;15(2):e0228308. doi: 10.1371/journal.pone.0228308 (PMC6996836; doi:10.1371/journal.pone.0228308)
Supplement: S3 Fig — (DOCX) [file pone.0228308.s003.docx]

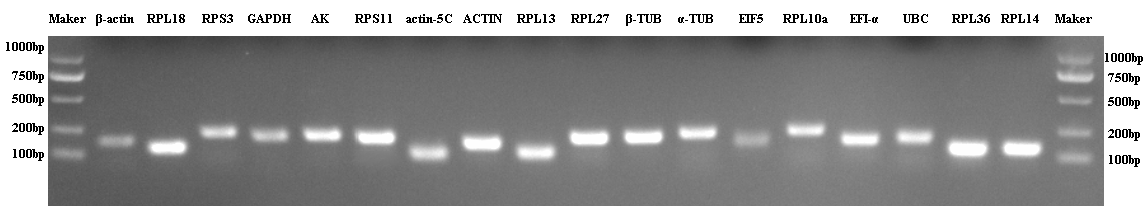


**Fig. S3 Agarose gel electrophoresis of RPC amplification products of ten candidate reference genes.**
