# Supplementary material for: Selection of reference genes for tissue/organ samples of adults of Eucryptorrhynchus scrobiculatus
Source: PLoS One. 2020 Feb 3;15(2):e0228308. doi: 10.1371/journal.pone.0228308 (PMC6996836; doi:10.1371/journal.pone.0228308)
Supplement: S1 Table — (DOCX) [file pone.0228308.s004.docx]

| gene | Primer sequence (5’-3’) | Tm(℃) | Product length (bp) | NCBI number |
| --- | --- | --- | --- | --- |
| β-actin | GTCTGTCTGGCAGTACAACCAC | 59 | 570 | MN706260 |
|  | CGACTTCGAACAGGAAATGGCC | 60 |  |  |
| RPL18 | GTCATTGGGCGCAAGTTGCC | 61 | 406 | MN706259 |
|  | CGTGGAATTGTTTGACTTGGGGC | 61 |  |  |
| RPS3 | GGGTCTTTCGAAAGGAGGC | 58 | 591 | MN706244 |
|  | GCCTTTTCAATGTCCTTGGCG | 59 |  |  |
| GAPDH | CTTATTCAAATACGATTCAACCCACGG | 58 | 756 | MN706245 |
|  | GCATCAAACACCGAGCTGTGAG | 61 |  |  |
| AK | ATGGTTGACGCCGCAGTTCTC | 61 | 1174 | MN706246 |
|  | GCAATAAGCAGTCTCCGCTTCTG | 60 |  |  |
| RPS11 | CGATGGCCACTACGTCTCC | 59 | 380 | MN706247 |
|  | TCACAGTGTCAACATGGCGG | 59 |  |  |
| Actin-5C | GTAATACTTTGGCCTAGTGCACATACG | 59 | 1277 | MN706248 |
|  | GCCGCATTAGTCGTAGACAATGG | 60 |  |  |
| ACTIN | GTCCGTGACATCAAGGAAAAACTTTG | 58 | 444 | MN706249 |
|  | CCACATCTGTTGGAAGGTGGAG | 58 |  |  |
| RPL13 | GCGAAAAGCCTATCCTGATCGAC | 59 | 450 | MN706243 |
|  | CCACTGACTGGTATTTCCAGCC | 59 |  |  |
| RPL27 | GGGTAAAATAATGAAGCAAGGCAAAGTC | 59 | 406 | MN706250 |
|  | CTAGAACCTCAATTTTTGGAAGAACCATTTG | 58 |  |  |
| β-TUB | TGAGGGAAATCGTTCACATCCAAGC | 60 | 1336 | MN706251 |
|  | TCATCGACTTCGCCTTCTTGGTC | 60 |  |  |
| α-TUB | GTTCGTTTGGGAAGCTGCATAACTAG | 60 | 1419 | MN706252 |
|  | CTTCTCCAGAGTCCATTCCTACTTCTTC | 59 |  |  |
| EIF5 | ATGAGCCTAAACGTAAACCGTAACG | 59 | 1335 | MN706253 |
|  | TTAGATTGCGTCAATGTCGACGTC | 58 |  |  |
| EF1-α | CCCGTCTCGTTAAGTTGTTAAATTTC | 57 | 1454 | MN706254 |
|  | CTGCAGATTTCGTTACTTTACCAGC | 58 |  |  |
| UBC  RPL36  RPL14 | CCTTCAGATACCATTGAAAACGTTAAAGC | 58 | 449 | MN706255 |
|  | CAAGTGTGATGGTTTTTCCTGTAAGGG | 59 |  |  |
| RPL36 | GAGCGGCAGCAGCTTTTC | 57 | 528 | MN706256 |
|  | CATGTCACTTTAATTAAATGGTTTAGTAGATGAC | 57 |  |  |
| RPL14 | ATGGCCTTCGAAAGGTTTGTGG | 60 | 450 | MN706257 |
|  | GCCGCCTTTGGCCATCTTC | 60 |  |  |
| RPL10a | ATGTCGTCGAAAGTTTCTAGGGATAC | 59 | 654 | MN706258 |
|  | ATGTCGTCGAAAGTTTCTAGGGATAC | 59 |  |  |

**Table S1.** Primers used for amplifying 18 reference genes in *E. scrobiculatus.*
