# Supplementary material for: Selection of reference genes for tissue/organ samples of adults of Eucryptorrhynchus scrobiculatus
Source: PLoS One. 2020 Feb 3;15(2):e0228308. doi: 10.1371/journal.pone.0228308 (PMC6996836; doi:10.1371/journal.pone.0228308)
Supplement: S2 Table — (DOCX) [file pone.0228308.s005.docx]

**Table S2. Primers used for amplifying and quantified GH45 in *E. scrobiculatus*.**

| Gene | Primer sequences (5’-3’) | Tm(℃) | Product length (bp) |
| --- | --- | --- | --- |
| CH45-amplifying | ATGAAAACCTTTATTGCCATTTCCG | 57 | 693 |
|  | CTAAAAAAAGTCTTCATTGTCGATTCCG | 57 |  |
| CH45*-* quantified | GAACGTGAACACTGGCGGAGATT | 60 | 172 |
|  | TGGAAGTTCGTTACACTCGGCATC | 59 |  |
