# Supplementary material for: Selection of reference genes for tissue/organ samples of adults of Eucryptorrhynchus scrobiculatus
Source: PLoS One. 2020 Feb 3;15(2):e0228308. doi: 10.1371/journal.pone.0228308 (PMC6996836; doi:10.1371/journal.pone.0228308)
Supplement: S3 Table — (DOCX) [file pone.0228308.s006.docx]

|  | β-actin | RPS3 | AK | GAPDH | RPS11 | RPL18 | actin-5C | ACTIN | RPL13 | RPL27 | β-TUB | α-TUB | EIF5 | RPL10a | EF1-A | UBC2 | RPL36 | RPL14 |
| --- | --- | --- | --- | --- | --- | --- | --- | --- | --- | --- | --- | --- | --- | --- | --- | --- | --- | --- |
| Malpighian tubule | 24.00493 | 22.71127 | 31.09666 | 27.16435 | 24.97834 | 24.93427 | 27.42755 | 24.7696 | 23.17014 | 23.28014 | 23.35176 | 25.41132 | 25.4774 | 19.80307 | 21.07262 | 23.89184 | 21.43706 | 21.82929 |
| hindgut | 22.97044 | 22.19074 | 26.95972 | 27.3828 | 24.30166 | 23.17464 | 23.88883 | 24.5654 | 22.5787 | 22.74579 | 22.38999 | 24.13554 | 26.31056 | 19.89191 | 21.45272 | 24.61745 | 21.3499 | 21.76657 |
| antenna | 24.60129 | 25.4231 | 29.72654 | 30.27462 | 27.59761 | 27.54461 | 24.34541 | 24.11943 | 25.64425 | 26.42842 | 25.875 | 25.87659 | 27.86578 | 23.73173 | 24.4625 | 26.89775 | 23.92632 | 25.08203 |
| head | 27.04672 | 31.30337 | 34.76908 | 35.69347 | 32.24364 | 32.28618 | 25.69331 | 26.73219 | 30.14322 | 30.57818 | 29.00834 | 31.49926 | 35.19321 | 30.64763 | 30.34337 | 33.8452 | 30.47573 | 30.65508 |
| foregut | 22.01167 | 24.87092 | 29.12815 | 28.04719 | 25.85473 | 26.2894 | 22.932 | 23.97869 | 24.84398 | 25.75834 | 25.68495 | 26.58272 | 27.77711 | 22.74764 | 24.14995 | 26.66394 | 23.81287 | 24.45357 |
| leg | 19.3824 | 23.4724 | 28.33721 | 28.12424 | 26.35085 | 25.5687 | 20.22724 | 21.11657 | 24.5568 | 25.04434 | 22.40771 | 24.67838 | 27.361 | 23.16709 | 24.69486 | 26.48297 | 22.38951 | 23.48689 |
| male genitalia | 24.6921 | 23.97136 | 30.47283 | 27.96115 | 26.15711 | 25.67876 | 28.11212 | 24.98251 | 24.01361 | 24.34504 | 23.35084 | 23.52948 | 21.15598 | 21.50297 | 24.28413 | 22.99696 | 22.40648 | 22.83476 |
| female genitalia | 17.35128 | 20.61291 | 25.25576 | 23.98615 | 22.92668 | 22.75744 | 17.43692 | 19.53816 | 20.91169 | 20.60252 | 20.40421 | 21.14826 | 24.80822 | 18.62709 | 20.14771 | 22.76332 | 20.06275 | 21.02076 |
| wing | 27.95466 | 25.43907 | 34.46736 | 31.46455 | 26.76591 | 26.75036 | 32.65994 | 26.83674 | 25.69868 | 26.44019 | 25.64223 | 26.23733 | 29.90828 | 23.25006 | 24.64207 | 27.20889 | 23.74699 | 23.7914 |
| midgut | 21.80881 | 22.99039 | 29.49659 | 25.1593 | 24.43258 | 24.12513 | 22.99275 | 22.99429 | 23.62174 | 23.41443 | 23.67006 | 25.59795 | 27.44353 | 21.51563 | 23.26323 | 25.82252 | 22.15369 | 22.19156 |

**Table S3 Average Ct values of 18 candidate reference genes.**
