# Supplementary material for: Selection of reference genes for tissue/organ samples of adults of Eucryptorrhynchus scrobiculatus
Source: PLoS One. 2020 Feb 3;15(2):e0228308. doi: 10.1371/journal.pone.0228308 (PMC6996836; doi:10.1371/journal.pone.0228308)
Supplement: S4 Table — (DOCX) [file pone.0228308.s007.docx]

**Table S4. Stability values of 18 candidate reference genes given by four algorithms.**

| **Delta CT** | | **NormFinder** | | **geNorm** | | **BestKeeper** | |
| --- | --- | --- | --- | --- | --- | --- | --- |
| Gene | Stability value | Gene | Stability value | Gene | Stability value | Gene | Stability value |
| actin-5C | 3.92 | actin-5C | 3.825 | actin-5C | 1.797 | actin-5C | 3.121 |
| EIF5 | 2.523 | EIF5 | 2.256 | β-actin | 1.532 | β-actin | 2.478 |
| β-actin | 2.44 | β-actin | 2.06 | ACTIN | 1.406 | GAPDH | 2.371 |
| ACTIN | 2.224 | ACTIN | 1.753 | EIF5 | 1.282 | EIF5 | 2.314 |
| AK | 2.047 | AK | 1.469 | AK | 1.141 | RPL10a | 2.22 |
| UBC | 1.731 | UBC2 | 1.336 | GAPDH | 0.996 | AK | 2.184 |
| GAPDH | 1.703 | RPL10a | 1.247 | UBC2 | 0.927 | UBC2 | 2.101 |
| RPL10a | 1.632 | EF1-A | 1.072 | α-TUB | 0.87 | RPL27 | 1.986 |
| EF1-α | 1.572 | GAPDH | 1.037 | β-TUB | 0.807 | RPS3 | 1.968 |
| α-TUB | 1.567 | RPL14 | 0.986 | RPL10a | 0.725 | β-TUB | 1.899 |
| β-TUB | 1.516 | α-TUB | 0.871 | EF1-A | 0.672 | EF1-A | 1.894 |
| RPL14 | 1.452 | RPL36 | 0.779 | RPL27 | 0.613 | RPL36 | 1.851 |
| RPS11 | 1.384 | β-TUB | 0.705 | RPL13 | 0.585 | RPL18 | 1.845 |
| RPL36 | 1.373 | RPS11 | 0.704 | RPS11 | 0.568 | RPL14 | 1.827 |
| RPL18 | 1.368 | RPL18 | 0.693 | RPL18 | 0.528 | α-TUB | 1.689 |
| RPL27 | 1.336 | RPS3 | 0.501 | RPL14 | 0.457 | RPS11 | 1.663 |
| RPS3 | 1.289 | RPL27 | 0.497 | RPS3/RPL36 | 0.375 | RPL13 | 1.659 |
| RPL13 | 1.277 | RPL13 | 0.358 |  |  | ACTIN | 1.648 |
